# Extractions of Oil from *Descurainia sophia* Seed Using Supercritical CO_2_, Chemical Compositions by GC-MS and Evaluation of the Anti-Tussive, Expectorant and Anti-Asthmatic Activities

**DOI:** 10.3390/molecules200713296

**Published:** 2015-07-22

**Authors:** Jian-Hong Gong, Yan-Li Zhang, Jin-Li He, Xiao-Ke Zheng, Wei-Sheng Feng, Xiao-Lan Wang, Hai-Xue Kuang, Chun-Ge Li, Yan-Gang Cao

**Affiliations:** 1 School of Pharmacy, Heilongjiang University of Chinese Medicine, Harbin 150040, China; E-Mails: jianhong863@163.com (J.-H.G.); hxkuang@hotmail.com (H.-X.K.); 2 School of Pharmacy, Henan University of Traditional Chinese Medicine, Zhengzhou 450046, China; E-Mails: zyl2013hnzy@163.com (Y.-L.Z.); hnzy_hnzy@126.com (J.-L.H.); zhengxk.2006@163.com (X.-K.Z.); wangxiaohnz@163.com (X.-L.W.); lichunhnz@126.com (C.-G.L.); caoyan_yxy@163.com (Y.-G.C.)

**Keywords:** *Descurainia sophia* seed oil, supercritical fluid extraction, central composite design, response surface methodology, chemical compositions, pharmacological evaluation

## Abstract

*Descurainia sophia* is widely distributed in China and is one of the most troublesome annual weeds. It has diverse medicinal usage. *D. sophia* has abundant oil, making it an important oil plant in China. The main goal of this study was to obtain the maximum yield of the oil by an optimal selection of supercritical fluid extraction parameters. According to the central composite design and response surface methodology for supercritical fluid extraction method, a quadratic polynomial model was used to predict the yield of *D. sophia* seed oil. A series of runs was performed to assess the optimal extraction conditions. The results indicated that the extraction pressure had the greatest impact on oil yield within the range of the operating conditions studied. A total of approximately 67 compounds were separated in *D. sophia* seed oil by GC-MS, of which 51 compounds represented 98.21% of the total oils, for the first time. This study was also aimed at evaluating the anti-asthmatic, anti-tussive and expectorant activities *in vivo* of *D. sophia* seed oil which supplied for further research on bioactive constituents and pharmacological mechanisms.

## 1. Introduction

Oil seeds of plants are usually extracted with organic solvents intended to remove the neutral lipids. In routine laboratory extraction methods, oil extraction requires a long time to complete and consumes significant amounts of solvents. However, for technological, economical and environmental safety issues, research is currently geared towards the development of alternative extraction procedures using environmentally-friendly technologies as well as other organic solvents [1,2,3].

Common gases, such as carbon dioxide (CO_2_), in their supercritical state have properties and extraction capacities very similar to liquids [4]. CO_2_ is the most widely used supercritical fluid (SFE) in food and pharmaceutical industry, because obtained extracts contain no organic residues. Furthermore, CO_2_ is non-toxic, non-flammable, chemically stable, inexpensive and has a low critical point of temperature and pressure [5]. Selection of the extraction conditions (pressure, temperature and time) can affect the final yield [6,7,8].

Optimization of the reaction parameters involved in an extraction method is commonly made by varying one factor at a time and keeping the others constant. However, this method is inefficient, as it fails to explain relationships between the variables and the response when there is interaction between the variables [9]. Response surface methodology (RSM), an effective statistical technique, can optimize complex extraction procedures by investigating the variables and the interactions of the variables simultaneously [10,11,12,13]. Usually, it applies an experimental design such as central composite design (CCD) to fit a second-order polynomial by a least squares technique. An equation is used to describe how the test variables affect the response and determines the interrelationship among the variables [14,15,16].

*Descurainia sophia* (L.) Webb ex Prantl (Flixweed), which originated in South Europe and North Africa, is a member of family *Brassicaceae*. It is widely distributed in northeastern China and is one of the most troublesome annual weeds, widely occurred in the major wheat planting regions of China. It is a vigorous competitor with prolific seed production, with the ability to quickly invade wheat fields [17,18]. *D. sophia* is also widely used in folk medicine and has diverse medicinal usage, such as a remedy for throat disease, measles, and smallpox [19]. In particular, the seeds of this plant have been used in Traditional Chinese Medicine (TCM) to relieve cough, prevent asthma, reduce edema, and promote urination [20]. Previous work has been undertaken on this plant that it contains various types of secondary metabolites, such as glucosinolates, cardiacglycosides, flavonoids, lactones, lipids, sulfur glycoside, orlignan, coumarins, allyl, and benzyl isothiocyanates and β-sitoterol with biological effects [19,20,21].

*D. sophia* seed oil was extracted exhaustively with benzine (70–80 °C) for extraction to afford a yellowish-brown oil in 22% yield [19]. However, so far there is no information published about the optimization of extraction conditions for oil from the seeds of *D. sophia*. In the present study, the process conditions have been optimized by RSM and a CCD (3 factors and 4 levels) for determining the factors effects and their interactions. The aims of our research are to investigate and optimize the most efficient SFE parameters (extraction pressure, extraction temperature and extraction time) on the extraction of *D. sophia* seed oil to achieve the highest extraction yields. This is part of an effort for the use of clean sample preparation techniques, with the aim to reduce the occupational exposure of the analysts to toxic solvents and their overall disposal to the environment by laboratories whose principal role should be to ensure environmental safety [22]. After that, gas chromatography-mass spectrometry (GC-MS) was used to analyze the chemical composition of *D. sophia* seed oil.

Importance of pharmacologically active natural compounds and plant sources has been reevaluated in recent years and it has become one of the most active research fields. We designed a series of experiments to evaluate the anti-tussive, expectorant and anti-asthmatic effects of activities of the extracted oil, trying to confirm its traditional use with scientific evidence and providing a theoretical basis for the commercial exploitation of *D. sophia* seed oil.

## 2. Results and Discussion

### 2.1. Statistical Analysis and the Model Fitting

Response surface optimization is more advantageous than the traditional single parameter optimization in that it saves time and raw material [23,24]. There were a total of 20 runs for optimizing the three individual parameters in the current CCD.

Three independent parameters in the study were namely extraction time, extraction pressure and extraction temperature as response (dependent variable). The ranges and the levels of the independent variables are given in Table 1. The extraction yields of *D. sophia* seed oil obtained under the twenty different testing conditions are shown in Table 2.

**Table 1:** Experimental range and levels of independent test variables.

Variables	Ranges and Levels				
	−1.68	−1	0	1	1.68
A (Pressure, MPa)	4.89	10	17.50	25	30
B (Temperature, °C)	43.18	50	60	70	76.82
C (Time, min)	2.50	40	95	150	187.50

Table 2 shows the experimental conditions and the results of extraction yield of *D. sophia* seed oil according to the factorial design. By applying multiple regression analysis on the experimental data, the response variable and the test variables were related by the following second-order polynomial equation:

Extraction yield = −15.85157 + 2.40593A + 0.30916B + 0.10309C − 0.019167AB + 0.000915152AC − 0.000581818BC − 0.027311A^2^ − 0.00134315B^2^ − 0.000226169C^2^ (1)

Extraction yield was the yield of *D. sophia* seed oil (%). A, B and C were the actual values of the variables for extraction pressure (MPa), temperature (°C) and time (min), respectively. The results of the analysis of variance, goodness-of-fit and the adequacy of the models were summarized in Table 3. The determination coefficient (R^2^ = 0.9836) was showed by ANOVA of the quadratic regression model, indicating that only 1.64% of the total variations were not explained by the model. The value of the adjusted determination coefficient (Adjusted R^2^ = 0.9689) also confirmed that the model was highly significant. At the same time, a very low value 2.52 of coefficient of the variation (CV) clearly indicated a very high degree of precision and a good deal of reliability of the experimental values. The model was found to be adequate for prediction within the range of experimental variables. The *p*-values were used as a tool to check the significance of each coefficient, which in turn may indicate the pattern of the interactions between the variables. The smaller was the value of P, the more significant was the corresponding coefficient [25,26]. It can be seen from this table that the linear coefficients (A, B, C), a quadratic term coefficient (A^2^, C^2^) and cross product coefficients (AB) were significant, with very small *p*-values (*p* < 0.05). The other term coefficients (AC, BC, B^2^) were not significant (*p* > 0.05).

The “Lack of Fit *F*-value” of 1.46 and the probability (*p*) value of 0.3443 (*p* > 0.05) imply that the Lack of Fit is not significant relative to the pure error, which measured the fitness of models, so the results indicated that the model was accurate for predicting response variations. In brief, the responses were explained well by the regression equation, and this allowed it to establish response surfaces and it was feasible to use the regression models to predict the yields of *D. sophia* seed oil. The full model developed from Equation (1) was used to make three-dimensional and contour plots to predict the relationships between the independent variables and the dependent variables.

**Table 2:** Experimental program and results for SFE–CO_2_ of *D. sophia* seed oil.

No	A Pressure	B Temperature	C Time	Pressure (MPa)	Temperature (°C)	Time (min)	Extraction Yield (%)
1	0	0	0	17.5	60	95	26.48
2	1.68	0	0	30	60	95	27.32
3	1	–1	–1	25	50	40	25.30
4	0	0	0	18	60	95	27.01
5	1	1	–1	25	70	40	25.69
6	0	0	0	18	60	95	26.49
7	0	0	0	18	60	95	27.25
8	–1	1	–1	10	70	40	22.25
9	0	1.68	0	18	77	95	28.78
10	0	0	0	18	60	95	28.01
11	0	0	–1.68	18	60	3	21.15
12	–1	–1	1	10	50	150	21.40
13	0	0	0	18	60	95	27.40
14	–1	–1	–1	10	50	40	18.15
15	1	1	1	25	70	150	29.17
16	–1.68	0	0	5	60	95	18.14
17	–1	1	1	10	70	150	26.26
18	1	–1	1	25	50	150	32.10
19	0	–1.68	0	18	43	95	26.13
20	0	0	1.68	18	60	187	29.13

**Table 3:** *D. sophia* seed oil extraction yield the regression equation coefficient and significant testing.

Source	Sum of Squares	df	Mean Square	*F* Value	*p*-Value Prob > *F*	
Model	252.16	9	28.02	66.83	<0.0001	significant
A-Pressure	115.05	1	115.05	274.42	<0.0001	
B-Temperature	8.66	1	8.66	20.66	0.0011	
C-Time	70.19	1	70.19	167.41	<0.0001	
AB	16.53	1	16.53	39.43	<0.0001	
AC	1.14	1	1.14	2.72	0.1302	
BC	0.82	1	0.82	1.95	0.1924	
A^2^	34.01	1	34.01	81.12	<0.0001	
B^2^	0.26	1	0.26	0.62	0.4493	
C^2^	6.75	1	6.75	16.09	0.0025	
Residual	4.19	10	0.42			
Lack of Fit	2.49	5	0.50	1.46	0.3443	not significant
Pure Error	1.70	5	0.34			
Cor Total	256.36	19				

Values of “Prob > *F*” less than 0.05 indicate model terms are significant.

### 2.2. Optimization of SFE Experimental Conditions

**Figure 1:**
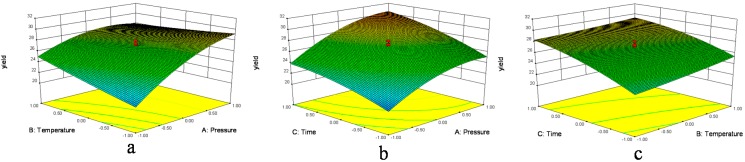
Response surface plots (3D) showing the effects of variables for the yield of *D. sophia* seed oil. (**a**) Effect of extraction pressure (A) and extraction temperature (B) under extraction time (C) 95 min; (**b**) effect of A and C at B = 60 °C and (**c**) effect of C and B at A = 17.5 MPa.

**Figure 2:**
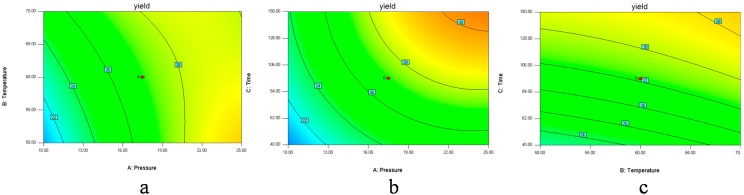
Contour plots (2D) showing the effects of variables (**a**) extraction pressure (A) and temperature (B); (**b**) extraction pressure (A) and time (C); (**c**) extraction temperature (B) and time (C) on the yield of *D. sophia* seed oil.

The graphical representations of the regression Equation (1), called the response surfaces and the contour plots, were obtained using Design-export 8.0, and the results of extraction yield of *D. sophia* seed oil affected by extraction pressure, extraction temperature and extraction time are presented in Figure 1 and Figure 2. Response surface methodology plays a key role in identifying the optimum values of the independent variables efficiently, under which dependent variable could arrive the maximum response [16]. In the response surface plot and contour plot, the extraction yield of *D. sophia* seed oil was obtained along with two continuous variables, while the other variable was fixed constant at its zero level (center value of the testing ranges). In the two figures, the maximum predicted value indicated by the surface was confined in the smallest ellipse in the contour diagram. Elliptical contours are obtained when there is a perfect interaction between the independent variables. The independent variables and maximum predicted values from the figures corresponded with the optimum values of the dependent variables (responses) obtained by the equations [27,28,29].

In Figure 1a and Figure 2a, when the 3D response surface plot and the contour plot were developed for the extraction yield of *D. sophia* seed oil with varying extraction temperature and extraction pressure at fixed extraction time, the extraction yield of *D. sophia* seed oil increased with the increasing extraction temperature, and increased rapidly with increase of extraction pressure from 10 to 17.5 MPa, then gradually decreased from 17.5 to 25 MPa. The Figure 1b and Figure 2b showed the 3D response surface plot and the contour plot at varying extraction pressure and extraction time at fixed extraction temperature. As in the case of the extraction yield of *D. sophia* seed oil, extraction pressure had a positive impact on the extraction yield of *D. sophia* seed oil. There was an increase in the extraction yield of *D. sophia* seed oil with increase in the extraction time. However, the extraction yield of *D. sophia* seed oil was found to increase rapidly with increase of extraction time from 40 to 95 min, but beyond 95 min, the yield increased slowly with increasing extraction. The results are in accord with the data in Table 3, which showed that the interactive effect of extraction pressure with extraction time on the yield of *D. sophia* seed oil was not very weak (*p* = 0.1302). The 3D response surface plot and the contour plot based on independent extraction temperature and extraction time are shown in Figure 1c and Figure 2c, while the other independent variable, extraction pressure at a zero level. An increase in the extraction yield of *D. sophia* seed oil could be evidently achieved with the increases of extraction temperature or extraction time. It was obvious that the extraction yield of *D. sophia* seed oil was almost proportional to extraction temperature and extraction time in certain range of variable.

Figure 1 and Figure 2 show the effects of pressure on the yield with each of the other two factors held constant. Pressure has the largest influence on the extraction yield. The extraction yields were increased with an increase in pressure, because raising the extraction pressure leads to a higher fluid density, which increases the solubility of the analytes. The solubility of oil in supercritical fluid directly affects the extraction yield and a balance between the SFE-CO_2_ density and the oil vapor pressure controls it. At high pressure, the influence of temperature on the solubility of oil is predominated by the oil vapor pressure effect, and then the solubility of oil increases with the increase of temperature. While at low pressure, SFE-CO_2_ density has a pronounced effect on the solubility of oil and the solubility decreases with the increase of temperature [10,30]. In Figure 1 and Figure 2, the graphs also show the effects of time with each of the two other factors on the yield. The trend was similar to the pressure. It may be that when the time reached a certain level the solubility reached saturation; the influence of the other factor temperature was not as significant as that of the pressure and time.

Optimization of extraction condition was achieved. It could be seen that the best extraction yield was reached at extraction pressure of 25 MPa, extraction temperature of 50 °C and extraction time of 150 min for the extractor and 3 MPa/40 °C for the separators. Among the three extraction parameters studied, extraction pressure was the most significant factor to affect the extraction yield of *D. sophia* seed oil, followed by extraction temperature, and extraction time according to the regression coefficients significance of the quadratic polynomial model (Table 3) and gradient of slope in the 3D response surface plot (Figure 1).

### 2.3. Verification of Predictive Model

The suitability of the model equations for predicting optimum response values was tested under the conditions: extraction temperature 50 °C, extraction time 150 min, and extraction pressure 25 MPa. This set of conditions was determined to be optimum by the RSM optimization approach and was also used to validate experimentally and predict the values of the responses using the model equation. The yield was measured by weighing this fraction after water removal by anhydrous sodium sulfate and by weight of *D. sophia* seed oil charged in the extractor. The isolated oils were yellowish liquids with a strong aromatic fragrance. That is, the mean value of extraction yield 31.79% (*n* = 3) with a relative standard deviation (RSD) of 1.029% was obtained from actual experiments, which demonstrated the validation of the RSM model and good agreement with the predicted value, so we may conclude that the model is satisfactory and accurate.

### 2.4. Chemical Compositions

GC-MS was used to analyze the chemical composition of *D. sophia* seed oil after methyl esterification which was found to have a very complex composition with compound identifications reported in Table 4. A total of approximately 67 compounds were separated in *D. sophia* seed oil, of which 51 compounds, representing 98.21% of the total oils, were positively identified by comparison of their retention indexes and the mass spectra of each GC component with those of standards and with reported data [31,32]. From GC analysis, 9, 12-octadecadiynoic acid methyl ester and methyl 10, 13, 16-docosatrienoate were found to represent 21.58% and 19.39% of the extracted oil, respectively. Other compounds identified in a large amount were γ-linolenic acid methyl ester (15.87%), isonicotinic acid, cyclohexyl ester (13.35%), dioctyl succinate (4.12%), 9-octadecynoic acid methyl ester (4.84%) and methyl 11,14-eicosadienoate (4.25%), and the remaining compounds, which number over 50 (15.06%).

There have been no reports regarding chemical composition analysis of *D. sophia* seed oil extracted by SFE-CO_2_. There have been a paper regarding GC analysis of oil composition of *D. sophia* seed oil which reported that 24 components were identified in *D. sophia* seed oil extracted exhaustively with benzine (70–80 °C) for extraction to afford a yellowish-brown oil in 22% yield [19]. Many of the fatty acids and other compounds present in oils have long been known to benefit our health. There is clearly great potential for developing functional oils. According to the results of this study, *D. sophia* seed oil has the amount of unsaturated fatty acid in the present study and *D. sophia* seed is an inexpensive source of essential fatty acid. For example, γ-linolenic acid (GLA) has been promoted as medication for a variety of ailments including breast pain and eczema [31]. GLA is also sometimes promoted as an anti-cancer agent. Neither GLA nor other GLA-rich supplements have been convincingly shown to be useful in preventing or treating any other health conditions [32,33,34,35]. 9-octadecynoic acid (oleic acid) is the most abundant fatty acid in human adipose tissue [36], and second in abundance in human tissues overall only to palmitic acid [37]. Oleic acid as its sodium salt is a major component of soap as an emulsifying agent. It is also used as an emollient [38]. Small amounts of oleic acid are used as an excipient in pharmaceuticals, and it is used as an emulsifying or solubilizing agent in aerosol products [39]. Oleic acid is also used to induce lung damage in certain types of animals, for the purpose of testing new drugs and other means to treat lung diseases [40]. Oleic acid is used as a soldering flux in stained glass work for joining lead came [41]. Therefore, *D. sophia* seed oil obtained with SFE method may have potential for use as a kind of specialty oil in various product applications. It is a cost effective technique at the laboratory scale and it seems to be applicable for industrial oil extraction.

**Table 4:** Identification and quantification of compounds contained in *D. sophia* seed oil after derivatisation compared with literature data.

No.	Compound		Relative Amount (%) ^a^	Molecular Formula	RI ^b^		RI ^c^
1	*cis-*1,2-Dihydrocatechol		0.01	C_6_H_8_O_2_	1060		1029
2	Carvol		0.01	C_10_H_14_O	1190		1154
3	1-Tridecene		0.01	C_13_H_26_	1292		1307
4	exo-2-Hydroxycineole acetate		0.01	C_12_H_20_O_3_	1344		1331
5	3-Dodecanone		0.01	C_12_H_24_O	1350		1331
6	Hydrocoumarin		0.03	C_9_H_8_O_2_	- ^d^		1370
7	Eugenol		0.03	C_10_H_12_O_2_	1362		1370
8	2-Dodecenal		0.01	C_12_H_22_O	1410		1432
9	Tetrahydroionol		0.01	C_13_H_26_O_4_	1432		1444
10	α-Ionone		0.01	C_13_H_20_O	1456		1444
11	*trans*-α-Bergamotene		0.01	C_15_H_24_	1430		1460
12	β-Sesquiphellandrene		0.01	C_15_H_24_	1446		1478
13	α-Farnesene		0.01	C_15_H_24_	1458		1478
14	Octylcyclohexane		0.03	C_14_H_28_	1476		1485
15	β-Bisabolene		0.01	C_15_H_24_	1500		1500
16	*trans*-Calamenene		0.02	C_15_H_22_	1537		1524
17	2,12-Dimethyltridecan-4-one		0.01	C_15_H_30_O	1589		1531
18	Prenyl salicylate		0.01	C_12_H_14_O_3_	-		1531
19	Vanillic acid		0.01	C_8_H_8_O_4_	1566		1547
20	Acetisoeugenol		0.01	C_12_H_14_O	1569		1599
21	7-Hexadecene		0.02	C_16_H_32_	1620		1633
22	1-Tetradecanol		0.01	C_14_H_30_O	1656		1637
23	β-Nootkatol		0.01	C_15_H_24_O	1662		1670
24	Benzoic acid, heptyl ester		0.03	C_14_H_20_O_2_	1682		1695
25	1-Nonylcycloheptane		0.01	C_16_H_32_	1696		1700
26	Heptadecane		0.19	C_17_H_36_	1711		1725
27	*n*-Pentadecanol		0.01	C_14_H_30_O_2_	1755		1739
28	(9*E*)-9-Octadecene		0.03	C18H36	1818		1825
29	Thymyl angelate		0.01	C_15_H_20_O_2_	-		1830
30	1-Hexadecanol		0.01	C_16_H_34_O	1854		1840
31		(*Z*)-11-Hexadecen-1-ol	0.01	C_16_H_32_O	-	1892		
32		Costunlide	0.42	C_15_H_20_O_2_	1897	1901		
33		Isonicotinic acid, cyclohexyl ester	13.35	C_12_H_15_NO_2_	-	1926		
34		*Z*-11-Hexadecenoic acid	0.01	C_16_H_30_O_2_	1976	1948		
35		Sulfurous acid dicyclohexyl ester	0.02	C_12_H_22_O_3_S	1964	1961		
36		Methyl 9,12-heptadecadienoate	0.02	C18H32O2	1994	1998		
37		3β,17β-Androstanediol	0.02	C_19_H_32_O_2_	-	2025		
38		(*Z*,*Z*)-9,12-Octadecadien-1-ol	0.02	C_18_H_34_O	2069	2060		
39		γ-Linolenic acid methyl ester	15.87	C_19_H_32_O_2_	2101	2091		
40		9,12-Octadecadiynoic acid methyl ester	21.58	C_19_H_30_O_2_	2112	2097		
41		9-Octadecynoic acid methyl ester	4.84	C_19_H_34_O_2_	2095	2099		
42		Methyl 11,14-eicosadienoate	4.25	C_22_H_40_O_2_	2118	2125		
43		*cis*-11, 14-Eicosadienoic acid methyl ester	10.11	C_21_H_38_O_2_	2292	2299		
44		Methyl 7, 11, 14-eicosatrienoate	1.6	C_21_H_36_O_2_	2300	2307		
45		Dioctyl succinate	4.12	C_20_H_38_O_4_	-	2335		
46		Butyl 9,12,15-octadecatrienoate	0.01	C_17_H_23_NO_4_	2399	2386		
47		Hydrastininic acid	0.04	C_11_H_9_NO_6_	2342	2400		
48		2-Chloroethyl linoleate	0.03	C_20_H_35_ClO_2_	2418	2432		
49		Methyl 10,13,16-docosatrienoate	19.39	C_23_H_42_O_2_	2499	2491		
50		Pentacosanal	1.18	C_25_H_50_O	-	2493		
51		*cis*-13,16-Docasadienoic acid	0.72	C_22_H_40_O_2_	2580	2531		

^a^ Percentages obtained by GC-FID peak-area normalization; ^b^ Retention indices calculated using an apolar column (DB–5); ^c^ Literature Retention indices using an apolar column (DB–5); ^d^ not detected.

### 2.5. Anti-Tussive, Expectorant and Anti-Asthmatic Activities

#### 2.5.1. Anti-Tussive Effects

The anti-tussive effects of *D. sophia* seed oil group on sensitive mice are shown in Table 5. Positive and *D. sophia* seed oil groups could enhance latent period of cough and the increase presented significantly. Compared to the control group, there was significant difference in positive and *D. sophia* seed oil group (*p* < 0.05) by ANOVA. Latent period of cough increased by 23.1%. *D. sophia* seed oil could inhibit cough frequency by 15.7%. Additionally, compared with cough frequency, the inhibitions presented significant difference (*p* < 0.05).

**Table 5:** Effect of *D. sophia* seed oil on the ammonia liquor induced cough in mice.

Group	Dose (mg∙kg^−1^)	Treatment	Latent Period (s)	Increasing (%)	Frequency	Inhibition (%)
control	-	ig	24.7 ± 4.76	-	30.4 ± 4.06	-
carbetapentane citrate capsules	125	ig	38.7 ± 6.18 **	56.7	18.9 ± 4.04 **	37.8
seed oil	1059	ig	30.4 ± 6.42 *	23.1	25.6 ± 4.77 *	15.7

Values expressed as mean ± S.E.M. (*n* = 10). * *p* < 0.05 for comparison of treated groups with control. ** *p* < 0.01 for comparison of treated groups with control.

#### 2.5.2. Expectorant Effects Secretion

The results of expectorant test were shown in Table 6. A standard curve of phenol red was obtained referring the Pharmacology Experiment Methodology, and the regression equation Y = 0.1387X + 0.0006 (Y = absorbance, X = the amount of phenol red secretion, *r* = 0.9998) was used. The results showed that positive and *D. sophia* seed oil could enhance tracheal phenol red output, compared with that of control. Besides, *D. sophia* seed oil had approaching expectorant effect (40.0%) in comparison with that of control. In addition, compared to the control group, there were significant differences for *D. sophia* seed oil group (*p* < 0.05) by ANOVA. These results indicated that the expectorant effects of *D. sophia* seed oil may be related to its ability to increase tracheobronchial mucus secretion and decrease the viscosity of mucus [42].

**Table 6:** Effects of *D. sophia* seed oil on tracheal phenol red output in mice.

Group	Dose (mg∙kg^−1^)	Treatment	Phenol Red Secretion (μg∙mL^−1^)	Absorbance (A)	Increasing (%)
control	-	ig	0.55 ± 0.14	0.08 ± 0.02	-
compound licorice tablets	650	ig	1.58 ± 0.28 **	0.22 ± 0.04 **	187.3
seed oil	1059	ig	0.77 ± 0.17 *	0.11 ± 0.02 *	40

Values expressed as mean ± S.E.M. (*n* = 10). * *p* < 0.05 for comparison of treated groups with control. ** *p* < 0.01 for comparison of treated groups with control.

#### 2.5.3. Anti-Asthmatic Effects

The effects of *D. sophia* seed oil, positive and control groups on sensitive guinea pigs exposed to mixture spray of 0.1% histamine and 2% acetylcholine chloride were shown in Table 7. It presented that *D. sophia* seed oil increased the preconvulsive time of asthma induced by the combination of histamine and acetylcholine chloride in guinea pigs at the dose of 528 mg∙kg^−1^ for five days. *D. sophia* seed oil group and positive group could enhance preconvulsive time by 162.1% and 36.3%, respectively. Compared to the control group, there were significant difference in *D. sophia* seed oil group (*p* < 0.05).

**Table 7:** Effect of the extract and fractions on guinea pigs bronchoconstraction induced by mixture spraying histamine and acetylcholine chloride.

Group	Dose (mg∙kg^−1^)	Treatment	Latent Period (s)	Increasing (%)
control	-	ig	87.67 ± 16.71	-
aminophylline tablets	100	ig	229.8 ± 36.23 **	162.1
seed oil	528	ig	119.5 ± 19.77 *	36.3

Values expressed as mean ± S.E.M. (*n* = 6). * *p* < 0.05 for comparison of treated groups with control. ** *p* < 0.01 for comparison of treated groups with control.

## 3. Experimental Section

### 3.1. Seed Collection and Preparation

*D. sophia* seeds were obtained from a herbal market in Zhengzhou, Henan provenance, China and identified by Prof. Chengming Dong, Henan University of TCM, China. Before analysis, they were stored in well-sealed containers at room temperature in a dark location. A voucher specimen (No. 20131102A) has been deposited in Department of Natural Medicinal Chemistry, Henan University of TCM.

### 3.2. D. sophia Seed Oil Extraction Procedures

A Suprex HA220-50-06 system (Jiangsu, China) in SFE mode with a maximal operating pressure of 50 MPa was used. The extraction vessel was a 1000 mL stainless steel vessel. A manual variable restrictor (Suprex) was used in the SFE system to collect the extracted analytes. The flow rate of supercritical CO_2_ (99.99% purity) (Zhengzhou, China) was approximately (0.4 ± 0.05 mL∙min^−1^). *D. sophia* seeds (500 g), was charged into the extraction vessel. The extracted analytes were collected in a 100 mL glass vial. After the completion of each run, the oil recovery percentage was calculated by weighing the collected solution.

### 3.3. Optimization Strategy

In the present study, response surface methodology (RSM) was applied to obtain the optimum experimental conditions providing the highest SFE recoveries of *D. sophia* seed oil inside the experimental domain. For this purpose, Design-Expert 8.05b application packages, statistical and graphical analysis software was employed to generate the experimental table and analysis the results [10]. *p*-values of less than 0.05 were considered to be statistically significant.

### 3.4. Derivation Process

The methyl esterification procedure was performed as follows: 10 mL of extract solution with 10 mL methanol and 2 mL H_2_SO_4_ was heated under reflux for 60 min at 65 °C. After cooling to room temperature, 10 mL hexane and 10 mL distilled water were added; the contents were shaken vigorously to mixed well and allowed to phase separate. The top hexane layer was transferred into a 10 mL vial. One milliliter of the organic phase, which was dried with anhydrous Na_2_SO_4_, was poured into a sample vial fitted with a 0.22-μm septum for GC-MS analysis [30].

### 3.5. The D. sophia Seed Oil Sample for GC-MS Analysis and Biological Activity Study

In the experiment, the chemical composition of a series of samples obtained by SFE has been analyzed by GC-MS. It showed that the higher the yield, the more different chemical components were obtained. The highest yield of the sample was used to carry out the analysis. That is, the *D. sophia* seed oil sample 18 (in Table 2) for GC-MS analysis and biological activity study was obtained from a 32.10% yield of *D. sophia* seed oil by an optimal selection of SFE parameters: extraction temperature 50 °C, extraction time 150 min, and extraction pressure 25 MPa. The *D. sophia* seed oil sample for GC-MS analysis of the samples was carried out through methyl esterification.

### 3.6. Gas Chromatography

*D. sophia* seed oils (0.1 μL), after methyl esterification, were injected neat into an a Agilent 7890 (Palo Alto, CA, USA) gas chromatography equipped with a 30 m × 0.25 mm HP-5 (cross-linked Phynel-methyl Siloxane) column with 0.25 μm film thickness (Palo Alto, CA, USA), was used for the study. Helium was used as carrier gas and the flow through the column was 2.25 mL∙min^−1^. The split mode with a split ratio of 1/50 was used. The oven temperature was as follows: 50 °C for 2 min rose from 50 °C to 200 °C at rate of 5 °C∙min^−1^ and finally raised from 200 °C to 300 °C at a rate of 5 °C∙min^−1^, and held at 300 °C for 3 min. The detector temperature was 280 °C. Injector temperature was held at 250 °C.

### 3.7. Mass Spectrometry Analysis

The oil after methyl esterification was analyzed by GC-MS using a Agilent 7000 mass selective detector coupled with a Agilent 7890A gas chromatograph (Palo Alto, CA, USA). The mass spectrometer scanned from 30 to 450 m∙s^−1^ with an ionization energy of 70 eV. Identification of components in the oil was based on retention indices relatives to n-alkanes from C_8_ to C_30_ was analyzed and computer matching with the NIST 05 library database and literature [43,44]. The chromatographic conditions were identical to those used for GC analysis. The percentage composition of compounds (relative quantity) in the oil was computed from the GC-FID peak areas using the normalization method, without correction factors.

### 3.8. Experimental Animals and Administration

Kunming mice of either sex (18–22 g) and guinea pigs of either sex (180–220 g) were purchased from Jinan (Shandong, China) (license number SCXKlu 20130001). All animals were housed at room temperature (18–22 °C) and provided food and water *ad libitum*. All animal experiments were performed according to the international rules considering animal experiments and the internationally accepted ethical principles for laboratory animal use and care.

After 3–5 days of adaptation, the eligible animals were randomly assigned to three groups and orally administered, including control group (distilled water), the medium dose of positive group (carbetapentane citrate capsules/125 mg∙kg^−1^, compound licorice tablets/650 mg∙kg^−1^, aminophylline tablets/100 mg∙kg^−1^ for anti-tussive, expectorant or anti-asthmatic experiment, respectively) (Compound licorice tablets are a Chinese patent medicine for expectorant) and the medium dose of *D. sophia* seed oil group (1059 mg∙kg^−1^ body weight for mice, 528 mg∙kg^−1^ for guinea pigs), which the dosage was based on 20 times human equivalent dose [45].

### 3.9. Anti-Tussive Effect Assessment

Anti-tussive activity was investigated on a classical mouse cough model induced by ammonia liquor. Briefly, each mouse was placed in a 4 L glass chamber and exposed to 25% NH_4_OH. The cough frequency and latent period of cough were recorded during 2 min exposure period. After 3 days of adaption, the mice were exposed to a 4 L glass chamber and sprayed with ammonium hydroxide (0.2 mL) to record the incubation time for 15 s and cough frequency. Mice with latent period less than 1 min and the cough frequency more than three times in 1 min were chosen to be eligible animals. The 30 eligible mice were divided into three groups (*n* = 10) randomly, including control, positive and *D. sophia* seed oil groups. The administration lasted for 7 days and the mice were exposed to the 4 L glass chamber with ammonium hydroxide (25%) after 30 min of the last administration and the cough incubation period was recorded. After 1 min, the mice were taken out from the glass chamber and placed in a beaker and the frequency of cough within 2 min was observed and recorded [46,47,48].

### 3.10. Expectorant Effect Assessment

The mice of either sex were randomly divided into three groups of 10 each. Experiments were carried out according to previously described methods [46,47,48,49]. Briefly, after the administration lasted for 7 days, each mouse was treated with a single dose (528 mg∙kg^−1^) of test drugs for 30 min before intraperitoneal injection of phenol red solution (5% in saline solution, *w*/*v*, 0.1 mL/10 g body weight). Thirty minutes after application of phenol red solution, mice were sacrificed by cervical dislocation without damaging the tracheas. The tracheas were dissected free from adjacent organs and removed from the thyroid cartilage to the main stem bronchi, then put into 2 mL normal saline immediately. After ultrasonic for 5 min, 0.1 mL of 1 M NaOH solution was added to the saline and optical density of the mixture were measured at 546 nm using Bio Mate 3S UV-vis spectrophotometer (Thermo Fisher Scientific, Waltham, MA, USA).

### 3.11. The Anti-Asthmatic Effect Assessment

To screen the sensitivity, guinea pigs were placed in a glass chamber (4 L) and sprayed with the mixture of 0.1% histamine and 2% acetylcholine chloride (1:1, *v*/*v*) under the average pressure of 400 ± 50 mmHg for 15 s [46,47,48]. The time to onset of respiratory distress and tumble (preconvulsive time) were recorded. The guinea pigs with preconvulsive time of more than 150 s were considered to be insensitive and discarded. The eligible guinea pigs were randomly allotted to three groups (*n* = 6) for control, positive and *D. sophia* seed oil groups (528 mg∙kg^−1^). All groups were treated with a single dose daily for 5 days and the last dose were given 1 h before the measurement of preconvulsive time. The delitescence of convulsion and tumble for each guinea pig within 6 min were observed. Guinea pig without convulsion and tumble was record as 360 s.

### 3.12. Statistical Analysis of Data

The experimental results of activities were expressed as the mean ± standard error of mean (S.E.M.) and analyzed using the software of Spss18. Values of *p* < 0.05 were considered to be significant between means of treated groups and control.

## 4. Conclusions

In conclusion, *D. sophia* seed oil was obtained with SFE method and the maximum yield of the oil by an optimal selection of SFE parameters. According to the CCD and RSM for supercritical fluid extraction method, a quadratic polynomial model was used to predict the yield of *D. sophia* seed oil from a fixed mass of seeds. A series of runs was performed to assess the optimal extraction conditions. The results indicated that the extraction pressure had the greatest impact on oil yield within the range of the operating conditions studied. A total of approximately 67 compounds were separated in *D. sophia* seed oil by GC-MS, of which 51 compounds represented 98.21% of the total oils, for the first time.

Through the pharmacological evaluation on ammonia induced mice coughing, intraperitoneal injection of phenol red in mice, together with histamine and acetylcholine chloride induced guinea pigs asthma, respectively, *D. sophia* seed oil appeared to be active for all of the assays. *D. sophia* seed oil either inhibited frequency or increased latent period of cough, which also supported anti-tussive effect of *D. sophia* seed. *D. sophia* seed oil fraction also showed significant expectorant effect *in vivo*. Additionally, *in vivo* evaluation with *D. sophia seed* oil fraction on the bronchoconstriction induced by mixed spray of histamine and acetylcholine chloride in guinea pigs, strongly supported the anti-asthmatic effect of them. The results supported for the use of *D. sophia* seed in the treatment of respiratory diseases. These effects are the important evidence for the traditional use of *D. sophia* seed as an anti-asthmatic remedy. The bioactive constituents and the mechanism of action and some structure-activity relationships among identified compounds explained for the observed activities have not been established, and thus further investigation should be conducted.
